# MxA mRNA Quantification and Disability Progression in Interferon Beta-Treated Multiple Sclerosis Patients

**DOI:** 10.1371/journal.pone.0094794

**Published:** 2014-04-14

**Authors:** Federico Serana, Luisa Imberti, Maria Pia Amato, Giancarlo Comi, Claudio Gasperini, Angelo Ghezzi, Vittorio Martinelli, Leandro Provinciali, Maria Rosa Rottoli, Stefano Sotgiu, Sergio Stecchi, Michele Vecchio, Mauro Zaffaroni, Cinzia Cordioli, Ruggero Capra

**Affiliations:** 1 CREA, Diagnostics Department, Spedali Civili of Brescia, Brescia, Italy; 2 Department NEUROFARBA, Neuroscience Section, University of Florence, Florence, Italy; 3 Department of Neurology, San Raffaele Scientific Institute, Milan, Italy; 4 Multiple Sclerosis Center, S. Camillo-Forlanini Hospital, Rome, Italy; 5 Multiple Sclerosis Center, Gallarate Hospital, Gallarate, Italy; 6 Neurological Clinic, Ospedali Riuniti of Ancona, Ancona, Italy; 7 Multiple Sclerosis Center, Papa Giovanni XXIII Hospital, Bergamo, Italy; 8 Department of Clinical and Experimental Medicine, University of Sassari, Sassari, Italy; 9 UOSI Riabilitazione Sclerosi Multipla, IRCCS Istituto delle Scienze Neurologiche of Bologna, Bologna, Italy; 10 Multiple Sclerosis Center, St. Elia Hospital, Caltanissetta, Italy; 11 Multiple Sclerosis Center, Spedali Civili of Brescia, Brescia, Italy; Institute Biomedical Research August Pi Sunyer (IDIBAPS) - Hospital Clinic of Barcelona, Spain

## Abstract

Even though anti-interferon beta (IFNβ) antibodies are the main determinants of IFNβ bioactivity loss and Myxovirus-resistance protein A (MxA) is the most established marker of IFNβ biological activity in IFNβ-treated multiple sclerosis patients, their usefulness in the routine clinical practice is still debated. Therefore, 118 multiple sclerosis patients naïve for treatment were enrolled for a 3-year longitudinal observational study mimicking the conditions of a real-world setting. In order to evaluate the kinetics of bioactivity loss in blood samples obtained every 6 months after therapy initiation, MxA and interferon receptor isoform/subunit mRNA were quantified by real-time PCR, anti-IFNβ binding antibodies were detected by radioimmunoprecipitation, and neutralizing antibodies by cytopathic effect inhibition assay. Clinical measures of disease activity and disability progression were also obtained at all time points. We found that, at the individual-patient level, the response to IFNβ therapy was extremely heterogeneous, including patients with stable or transitory, early or late loss of IFNβ bioactivity, and patients with samples lacking MxA mRNA induction in spite of absence of antibodies. No interferon receptor isoform alterations that could explain these findings were found. At the group level, none of these biological features correlated with the measures of clinical disease activity or progression. However, when MxA mRNA was evaluated not at the single time point as a dichotomic marker (induced vs. non-induced), but as the mean of its values measured over the 6-to-24 month period, the increasing average MxA predicted a decreasing risk of short-term disability progression, independently from the presence of relapses. Therefore, a more bioactive treatment, even if unable to suppress relapses, reduces their severity by an amount that is proportional to MxA levels. Together with its feasibility in the routine laboratory setting, these data warrant the quantification of MxA mRNA as a primary tool for a routine monitoring of IFNβ therapy.

## Introduction

Interferon beta (IFNβ) is widely used as first-line treatment for patients with relapsing remitting multiple sclerosis (MS). Three forms of IFNβ are currently available: intramuscular IFNβ-1a, subcutaneous IFNβ-1a, and subcutaneous IFNβ-1b. Although the formulation, frequency of administration, and dosage differ, all the IFNβ products are capable of reducing relapse rate by about 30% and new MRI lesions by about 70% [1]. Effectiveness appears to vary from patient to patient, with some of them achieving a robust treatment response and others that respond poorly, and continue to have clinical relapses, disability progression or active lesions on MRI. In addition, treatment regimens that require regular injections can be burdensome, which—together with their incomplete effectiveness—might lead some patients to poor long-term compliance [2].

The heterogeneous responses and the variable compliance represent a theoretical opportunity for a rational and personalized use of this drug, but the optimal marker of treatment response has still to be found, and the monitoring strategies and thresholds for therapy switch are not thoroughly defined. Accordingly, clarifying the response to treatment in individual patients with MS is notoriously difficult [3], [4], and several different markers have been proposed as potential indicators of IFNβ therapy success. In the field of MRI, evidence has now accumulated to show that the development of new lesions within 6–24 months after initiating IFNβ predicts an unfavourable response to this treatment and can help to identify patients with a poor prognosis [5], [6]. Among the biomarkers, which were analyzed at the protein and/or mRNA level, so far only neutralizing antibody (NAb) titers and IFNβ biological activity loss, measured by Myxovirus-resistance protein A (MxA) mRNA quantification, have proven clinically reproducible to some degree [5]. However, some disagreement still remains, in particular on the real role of NAb in predicting the therapeutic efficacy of IFNβ [7]–[9], also due to inter-laboratory variations between NAb assays [10], [11]. While, in general, the majority of the studies have been designed in a longitudinal fashion as far as the clinical and MRI data acquisition are concerned, the biological information regarding IFNβ bioactivity has been either collected from different patients analyzed at single time points, or retrospectively or based on randomized clinical trials, lacking the information contained in longitudinal data [12]. As a consequence, little evidence is available at the individual-patient level. Therefore, to analyse IFNβ bioactivity modulation in individual patients in the conditions of a real-life setting, we designed a 3-year prospective longitudinal study that was performed in subjects naïve for treatment initiating IFNβ therapy at the time of study inclusion. The primary outcome was the analysis of the kinetics of IFNβ bioactivity loss, defined according to MxA mRNA induction, and of anti-IFNβ antibody production. Secondary objectives were: to evaluate whether the expression of the mRNA for the IFNβ receptor (IFNAR) subunits and isoforms had a relevant impact on bioactivity loss; and to correlate the markers of IFNβ bioactivity with the measures of clinical disease activity, to determine whether biomarkers can predict IFNβ therapy effectiveness.

## Methods

### Patients

For this prospective longitudinal observational study, 118 patients (36 men and 82 women, between 18 and 64 years of age) with a diagnosis of relapsing–remitting MS according to the McDonald criteria [13] were consecutively enrolled. To be included, patients were required to have an Expanded Disability Status Scale (EDSS) ranging from 0 to 4.5 and to be naïve for IFNβ therapy. After enrolment, they received either intramuscular or subcutaneous (44 µg) IFNβ-1a (42 and 40 patients, respectively) or IFNβ-1b (36 patients), according to the principles of good clinical practice. The study lasted 36 months; visits were performed at baseline (T0), and then at 3 (T3), 6 (T6), 12 (T12), 24 (T24), 30 (T30) and 36 (T36) months of therapy. Blood was drawn by the multiple sclerosis center nurse only after the neurologist had verified compliance with the required time interval (12 hours [±1 hour] after the patient-declared timing of the last IFNβ injection). Patients were not receiving steroid therapy nor showed signs of viral infection at the moment of blood draw. At all time points, standard neurological assessments, including reporting of relapses and careful EDSS evaluation, were also required. After the end of the study, EDSS calculation was validated by a neurostatus level-C certified neurologist (http://www.neurostatus.net/).

### Ethics Statement

The study was approved by the ethical committee of Spedali Civili of Brescia (resolution n. 0863 of 12-20-2006), and all patients signed a written informed consent.

### MxA, BAb and NAb quantification

IFNβ bioactivity analysis was performed by a real-time reverse transcriptase PCR assay that measures MxA mRNA expression in patients' whole blood samples, as previously described [14]. Accordingly, the level of MxA mRNA induction was expressed as relative units also called “normalization ratio” (NR), which represents the fold-change in respect to MxA mRNA expression in a standard sample of a healthy donor. BAb and NAb quantification were performed as previously described, using a radioimmunoprecipitation assay for BAbs [14], and a cytopathic effect (CPE) inhibition assay for NAbs [15]. For BAb analysis, in order to correct for the different amount of total radioactivity obtained after each radiolabeling session, the count per minute read for each serum was normalized as the percentage of the total activity of that session; thus, the reported BAb levels are expressed as “% total activity”. BAb and MxA quantification was done in all patients at all time points, exception made for MxA, which was not done at T3; NAb titer was analyzed at several time points in selected samples (149 determinations from 84 patients). The levels of NAbs were expressed as ten-fold reduction units (TRU) [15].

### Quantification of IFNAR subunits and isoforms

Primers and probes for IFNAR2 and IFNAR2.2 were from Vitale et al. [16], while those for IFNAR1, IFNAR2.1 and IFNAR2.3 mRNA expression were designed with Primer Express software version 3.0 (Applied Biosystems) and were reported in Serana et al. [17], together with the employed real-time PCR protocol. The NR was used to express the results of IFNAR mRNA expression.

### Statistical analysis

The longitudinal analysis comparing the means between the groups of patients treated with the three drugs over the study period was performed by ANOVA for repeated measures based on linear mixed models, which were fitted with a random intercept. These models also allowed us to control for covariates, as well as to use all available data despite patients were progressively dropping-out (“unbalanced design”), thus enhancing the power of the analysis and providing a less biased picture of changes over time. For these analyses, the values of MxA, IFNAR and BAb were log-transformed in order to obtain an approximately normal distribution. Median EDSS changes between subgroups were compared by the Kruskal-Wallis and Mann-Whitney tests (used also to compare the area under the curve of non-transformed MxA values); annualized relapse rates were analyzed by negative binomial regression; proportions were compared by the Fisher's exact test. To identify factors associated to the risk of disability progression, logistic multivariable regression was performed. Several covariates including the age, basal EDSS, type of IFNβ treatment, presence of at least one relapse at T24, arithmetic mean of all log_2_-transformed MxA levels measured between T6 and T24, BAb levels, and IFNAR expression levels, were first tested as possible predictors in univariate fashion. When p<0.10, variables were retained and tested (together with their interactions) in multivariable models. The chosen model included as predictors the presence of at least one relapse at T24 and the “average” log_2_ MxA levels between T6 and T24 (their interaction was not significant and thus excluded). Based on this model, and considering that a back-transformation of the arithmetic mean of log-values corresponds to the geometric mean of the original values, the predicted probability of experiencing a disability increase after 2 years in relation to the geometric mean of non-transformed MxA NR values was calculated. P-value threshold was set at the level of 0.05.

## Results

### Study population

To perform a reliable longitudinal analysis of IFNβ bioactivity over the study period, we evaluated only patients with at least two consecutive laboratory determinations for each marker after therapy beginning. Therefore, 18 patients who left the study before T12 and had undergone only one MxA determination after therapy initiation were considered early dropouts and thus excluded from the analysis. Of the remaining 100 patients, 17 terminated the study before reaching T24, and 26 additional patients did not complete the study extension at T36, yielding a total dropout count of 43 subjects, which, added to the early dropouts, makes a total of 61 dropouts (52%), with 57 subjects (48%) completing the 3-year study period. Reasons for dropouts are listed in Table 1 and in the legend of Figure S1, and baseline characteristics of analyzed patients in Table S1.

**Table 1:** Dropout distribution.

Dropout for^a^	Early (<T12)^b^	Before T24	Before T36	Total
Adverse event	7	4	-	**11**
Disease worsening	4	8	2	**14**
Investigator decision	1	1	11	**13**
Lost to follow-up	-	2	6	**8**
Non-compliance	1	-	1	**2**
Consent withdrawal	5	2	6	**13**
	**18**	**17**	**26**	**61**

a See supplementary Figure S1 for more details.

b Early dropouts were excluded from the analysis because only one post-therapy MxA determination was available.

T12, T24, T36: 12, 24, 36 months post-therapy initiation.

### MxA, BAb, and NAb levels during treatment

**Figure 1:**
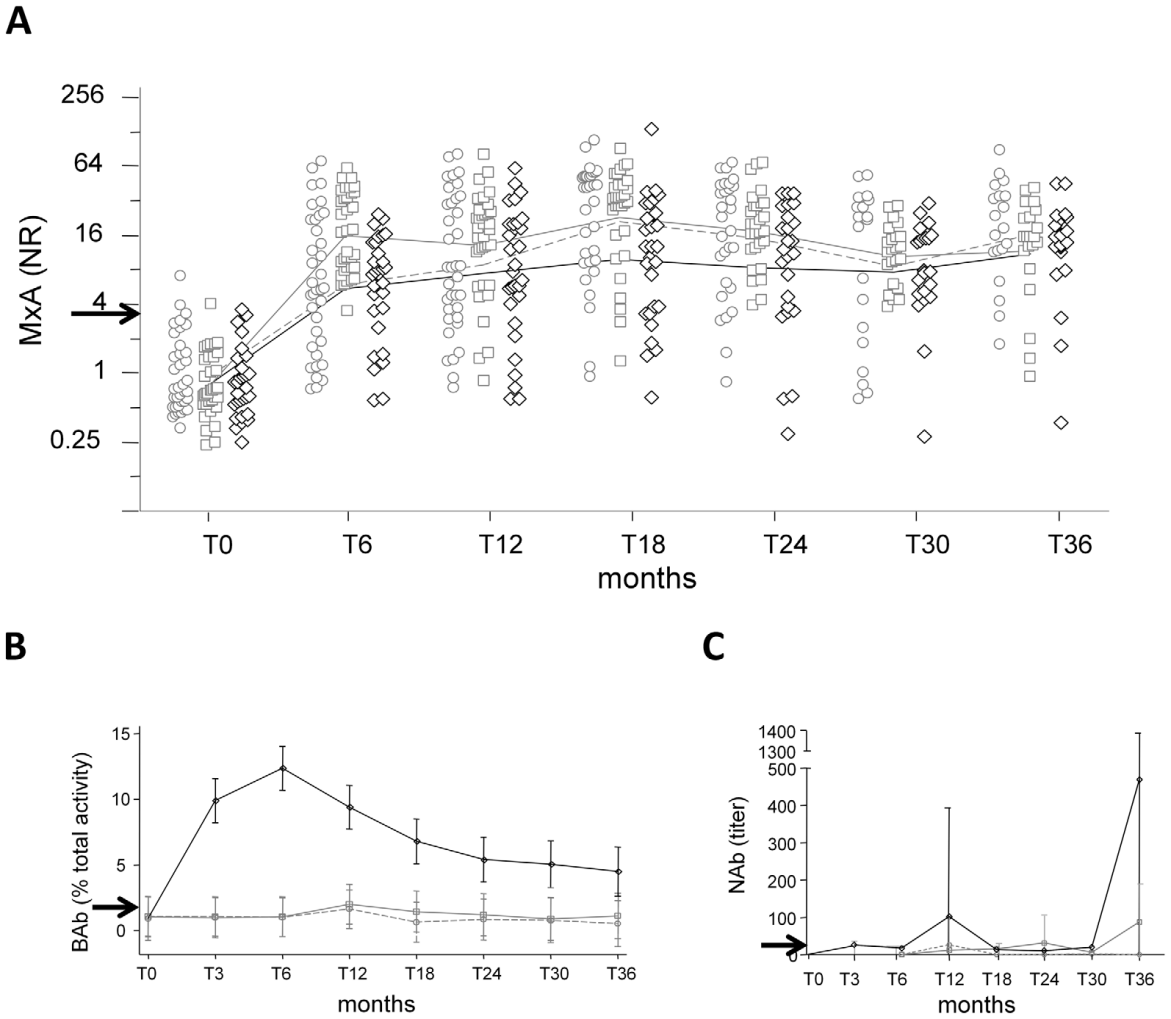
Kinetics of change of MxA, BAbs, NAbs during the study period. MxA values (A) in patients treated with IFNβ-1a i.m. (clear circles, dashed grey line), IFNβ-1a s.c. (clear squares, solid grey line), IFNβ-1b s.c. (clear diamonds, solid black line). Average kinetics of BAb production in all samples (B) and of NAb (C) production in selected samples in patients treated with IFNβ-1a i.m. (dashed grey line), IFNβ-1a s.c. (solid grey line), IFNβ-1b s.c. (solid black line). Lines connect predicted means. Arrows indicate the cut-offs. For BAbs, 95% confidence intervals are shown; for NAbs, standard deviations are shown because confidence intervals were not calculated (no statistical inference was performed due to selection bias). MxA: myxovirus-resistance protein A; NR: normalization ratio; BAbs: binding antibodies; NAbs: neutralizing antibodies; IFNβ: interferon beta.

The median MxA mRNA level at baseline was similar to that observed in a group of 100 healthy donors (0.78 NR; IQR:0.78–1.34 NR vs. 0.78 NR; IQR: 0.46–1.44 NR; p = NS) [18].Therefore, these MxA values were pooled together in order to determine the 99th percentile (3.83 NR), which was considered the cut-off used to classify patients as MxA mRNA induced (i.e. above the cutoff, MxA^+^) or MxA mRNA non-induced (i.e. below the cut-off, MxA^−^). After therapy initiation, the level of MxA mRNA increased similarly in the three treatment groups, and stayed constantly elevated, with minimal fluctuations, over the study period (Figure 1A). The only significant differences were observed at T6, when the MxA levels increased in patients receiving IFNβ-1a 44 µg s.c., and at T18, when MxA decreased in those receiving IFNβ-1b in comparison to the other two treatment groups.

Even if patients were naive for therapy, the levels of BAbs in the samples of MS patients obtained at T0 were significantly higher than that found in 140 healthy donors (median of % total activity: 0.98% vs. 0.62%; p<0.0001. Figure S2). Because this probably reflects the polyclonal increase of antibody production in MS, only the distribution of BAb values of MS patients at T0 was used to calculate the 99th percentile, which represented the cut-off for BAbs (% total activity:1.73%). The analysis of the kinetics of BAb production showed that the average BAb level during the course of treatment was higher in patients receiving IFNβ-1b in comparison to that of patients receiving IFNβ-1a (p<0.001 at all time points from T3 to T30), with a sharp and quick increase starting as early as 3 months after therapy initiation, a peak at T6, and then a slow decrease over the rest of the study period until reaching levels similar to those seen in IFNβ-1a-treated patients at T36 (Figure 1B). This average decrease, with a pattern highly similar to what previously shown by Lampasona et al. [19], was not only due to the increasing number of dropouts during the study period, because only three BAb-positive (BAb^+^) patients left the study between T6 and T24, which was the period characterized by the sharpest fall of BAb levels. Considering all samples pooled together, the sensitivity of BAb measurement in respect to detecting MxA^−^ samples was 46%, while the specificity was 75%.

When the CPE assay was performed, a sample was considered NAb-positive (NAb^+^) if it had a titer of at least 20 TRU, according to Antonelli et al. [15]. Because NAb levels were assayed only at selected time points, it was not possible to infer statistically tested conclusions. However, because NAb quantification was performed at least once for each patient, and more than once in many of the patients in whom the other two tests suggested a likely loss of bioactivity, a descriptive picture of the results can be drawn, indicating that the kinetics of NAb appearance was similar to that of BAbs, with an early rise, in particular in subjects treated with IFNb-1b (Fig 1C).

The two classes of anti-IFNβ antibodies were only partially overlapping because while NAbs were absent in all BAb-negative (BAb^−^) samples that were tested, they were found in only 32% of BAb^+^ patients, most of whom (90%) were treated with IFNb-1b (Figure S3).

### mRNA expression of IFNAR subunits/isoforms

**Figure 2:**
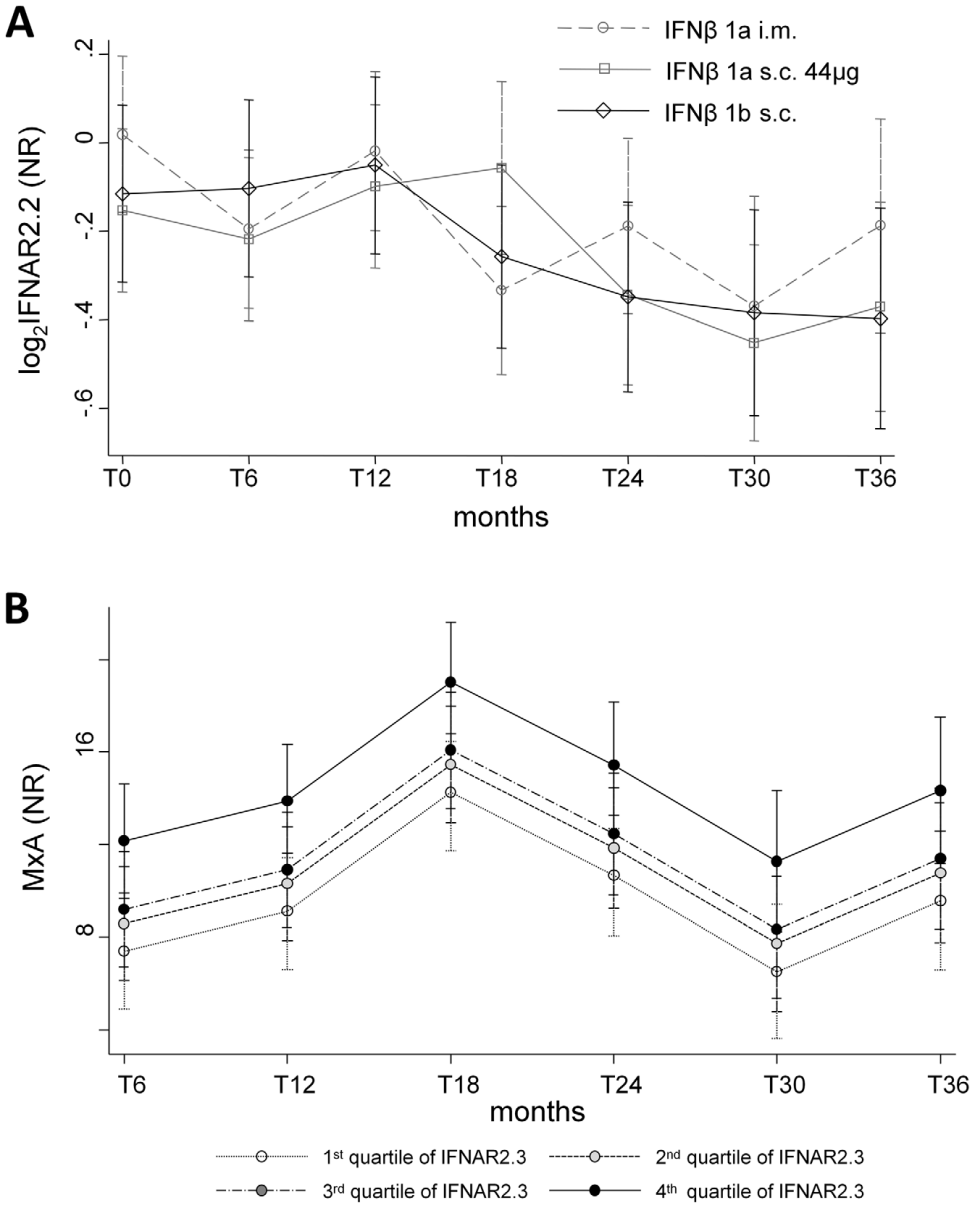
Role of IFNAR2.2 and IFNAR2.3 isoforms on IFNβ bioactivity. Average decrease of IFNAR2.2 mRNA (A) in patients treated with IFNβ-1a i.m. (clear circles, dashed grey line), IFNβ-1a s.c. (clear squares, solid grey line), IFNβ-1b s.c. (clear diamonds, solid black line). Lines connect predicted means; 95% confidence intervals are shown. Average change in MxA mRNA induction over time in dependence of different levels of expression of IFNAR2.3 mRNA (B), as determined by multivariable mixed-model regression. IFNAR: interferon receptor; IFNβ: interferon beta;MxA: myxovirus-resistance protein A; NR: normalization ratio.

In order to test whether IFNAR subunits modulation could have a significant impact on IFNβ bioactivity, especially after 3 years of continuous receptor stimulation, as suggested in the case of the subunit IFNAR1 [17],[20], and of the isoforms IFNAR2.2 and IFNAR2.3 [21], [22], we quantified the mRNA expression of all IFNAR subunits and isoforms at all time points. The only significant effect was a global decrease in IFNAR2.2 mRNA over time (Figure 2A), independently of the type of IFNβ administered. However, a multivariate regression model, performed after correcting for the presence of anti-IFNβ antibodies and type of IFNβ received, showed that, on average, the MxA mRNA levels were not influenced by modifications in the level of the isoform IFNAR2.2, similarly to what reported by Gilli et al. [22], but, unexpectedly, they increased with the growth of the soluble isoform IFNAR2.3 (Figure 2B).

### Analysis of the bioactivity profile at the individual-patient level

The kinetics of MxA mRNA induction, of BAb and NAb (when available) production was evaluated at the individual-patient level. A conserved IFNβ bioactivity would require the simultaneous presence of MxA-induction and, preferably, absence of BAbs and NAbs, according to the aforementioned cut-offs. Thus, the potentially “full biological responders” to IFNβ therapy would be those patients with a conserved IFNβ bioactivity at all time points. However, several intermediate situations (i.e. sporadic MxA non-inductions or isolated antibody positivity) and changes of bioactivity status were observed in the distinct patients during the study period. Therefore we defined categories that could represent the observed spectrum of bioactivity, which allowed us to classify patients into more homogeneous subgroups (Table 2).

**Table 2:** Characteristics of the patients and classification according to IFNβ-bioactivity profiles.

Bioactivity profile over the follow-up	Laboratory features	#	IFNβ-1a i.m. (#)	IFNβ-1a s.c. (#)	IFNβ-1b s.c. (#)
**Biological responder patients**	**MxA+BAb−NAb−**	**63**	**33**	**28**	**2**
	- constantly MxA+	37	13	22	2
	- with 1/2/3 sporadic MxA−	19/4/3	14/3/3	5/1/-	-
**Biological responder patients with BAbs**	**MxA+BAb+NAb−**	**24**	**1**	**5**	**18**
**Biological non-responder patients**	**MxA−**	**10**	**2**	**1**	**7**
- with anti-IFNβ antibodies	BAb+Nab+	8	-	1	7
- without anti-IFNβ antibodies	BAb−NAb−	2	2	-	-
**Patients with intermediate features**	**MxA/BAb/NAb +/−**	**3**	**1**	**-**	**2**
***TOTAL***		***100***	***37***	***34***	***29***

IFNβ: interferon beta.

MxA: myxovirus-resistance protein A.

BAb: binding antibodies.

NAb: neutralizing antibodies.

*A first group* of 63 biological responders (63%) was made up by 37 patients whose samples were MxA^+^, BAb^−^, NAb^−^ at all time points, and by 19 individuals that had only one isolated, sporadic non-induced MxA sample, in the context of a profile of high MxA-induction and constant lack of antibodies. Of note, all of these were being treated with IFNβ-1a (14 with IFNβ-1a i.m and 5 with sc IFNβ-1a). The 7 remaining patients of this group (all but one treated with IFNβ-1a i.m.), who presented with two or three non-consecutive, sporadic MxA non-inductions, were still considered biological responders because of the intermittent nature of their MxA non-induction in the total absence of NAbs or other factors, suggesting a truly significant loss of IFNβ bioactivity.

*A second group* of likely biological responders was made by 24 patients that were characterized, for the most part, by a conserved MxA induction, despite the presence of antibodies of the BAb class in at least four of the seven time points analyzed, with totally absent or below-the-cut-off (<20 TRU) NAbs. In particular, MxA mRNA was induced at all time points regardless of BAbs in 10 patients, while one MxA non-induced sample was found in 14 of them. In most patients BAbs appeared as early as 3 months after therapy beginning; peaked at the subsequent time point; and then gradually declined up to undetectable values at the end of the follow up, so that only 7 patients had BAbs at T36. Eighteen patients belonging to this group were treated with IFNβ-1b.

*A third group* of 10 patients was considered biological non-responder due to several consecutive MxA non-inductions, and was further divided into two subcategories because of the heterogeneity in the seeming reasons of bioactivity loss (presence vs. absence of anti- IFNβ antibodies). Indeed, *the first subcategory* was made by 8 patients whose samples resulted not only MxA-non-induced in two or more consecutive occasions, but were also simultaneously BAb^+^ and NAb^+^; however, NAbs, which were tested at several time points, resulted high (>200 TRU) only in 1 patient and very high (>400 TRU) in another one, and in their remaining samples their level of MxA induction was just above the cut-off or stayed fairly low (4<MxA<16 NR). These biological non-responders had been treated with the highest dose of IFNβ (7 with IFNβ-1b and 1 with IFNβ-1a 44 µg s.c). *The second subcategory* of biological non-responders was a puzzling subgroup of 2 patients treated with IFNβ-1a i.m who were consecutively MxA-non-induced at nearly all time points of the study period, in the absence of both BAbs and NAbs. Any altered IFNAR subunit/isoform expression that could explain this seeming bioactivity loss was not found (data not shown). In order to exclude the presence of genetic polymorphisms that could have rendered the MxA assay falsely negative, we have also sequenced the area bound by the real-time PCR MxA primers and probe, without finding any differences from the reference sequence (not shown). The kinetics of bioactivity loss was peculiar in at least 4 of the 10 biological non-responders: in 1 patient the bioactivity loss appeared late in the course of treatment (after 30 months); in 2 patients treated with IFNβ-1b both BAbs and NAbs disappeared and MxA reverted back to induction at T24 or at T30, suggesting that a late recovery of bioactivity is possible; also in 1 of the patients who had been repeatedly MxA-non-induced (but BAb^−^/NAb^−^), MxA reverted back to induction, albeit at a low level, at T36.

Finally, it was identified *a fourth group* of 3 patients, 2 of whom treated with IFNβ-1b and 1 with IFNβ-1a i.m., who showed intermediate features and could not be included in any of the previous schemes. Indeed, their MxA, even if sometimes low, was always above the cut-off, despite they had BAbs in at least two consecutive samples, and they had both BAbs and NAbs at one time point. BAbs and NAbs, however, declined to levels below the cut-off at the following time points, even if in 1 of these patients NAbs were high-titered (>200 TRU). This suggests that bioactivity, if not fully, was conserved for the most part of the study period.

### MxA and disability progression

The annualized relapse rate and the proportion of relapse-free patients after 24 or 36 months of study period were calculated and used as markers of disease activity, while an EDSS increase of at least 1 point over a period of at least 2 years was considered a significant marker of disability progression [23], [24]. As expected, the proportion of relapse-free patients after 2 years of treatment was significantly higher among those who did not show disability progression in the same time lapse (88% vs. 12%, p<0.001). However, no significant associations were found between these clinical parameters and the presence of BAbs or the absence of MxA induction, even if patients were grouped according to the different bioactivity profiles obtained by the individual-patient level analysis, or if groups were pooled together to compare all biological responder vs. all biological non-responder patients. The only exception was a significantly different (between the four groups) proportion of subjects with an increased EDSS after 2 years, which was likely due to the 2 biologically non-responder patients without antibodies, who both showed a clinical worsening (Table 3). These results suggest that the risk of disease evolution is not clearly predicted by classifying of patients on the basis of the MxA induction and the presence of antibodies.

**Table 3:** Markers of disease activity/progression in the patients with the different IFNβ-bioactivity profiles.

	Bioactivity profile over the follow-up					p-values	
	Biological responders	Biological responders with BAbs	Biological non-responders	Biological non-responders without Abs	Patients with intermediate features	Comparison between groups^a^	Pooled responders vs non-responders
**# pts reaching T24**	**50**	**20**	**8**	**2**	**3**		
% relapse-free at T24	74.00%	85.70%	62.50%	0.00%	100%	*0.08*	*0.08*
ARR T24 (mean)	0.02	0.07	0.37	0.50	0.00	*0.30*	*0.08*
% with ΔEDSS≥1 at T24	8.00%	14.30%	12.50%	100%	0.00%	*0.02*	*0.11*
ΔEDSS T0-T24 (median)	0.00	0.00	0.00	2.00	−0.17	*0.06*	*0.87*
**# pts reaching T36**	**32**	**17**	**4**	**1**	**3**		
% relapse-free at T36	71.90%	76.50%	50.00%	0.00%	100.00%	*0.36*	*0.15*
ARR T36 (mean)	0.10	0.06	0.33	0.33	0.00	*0.31*	*0.07*
% with ΔEDSS≥1 at T36	12.50%	11.80%	0.00%	100.00%	0.00%	*0.29*	*0.52*
ΔEDSS T0-T36 (median)	0.00	0.00	−0.25	1.00	0.00	*0.20*	*0.53*

a excluding the group of patients with intermediate features.

BAb: binding antibodies.

Abs: antibodies.

ARR: annualized relapse rate.

ΔEDSS: variation in Expanded Disability Status Scale.

**Figure 3:**
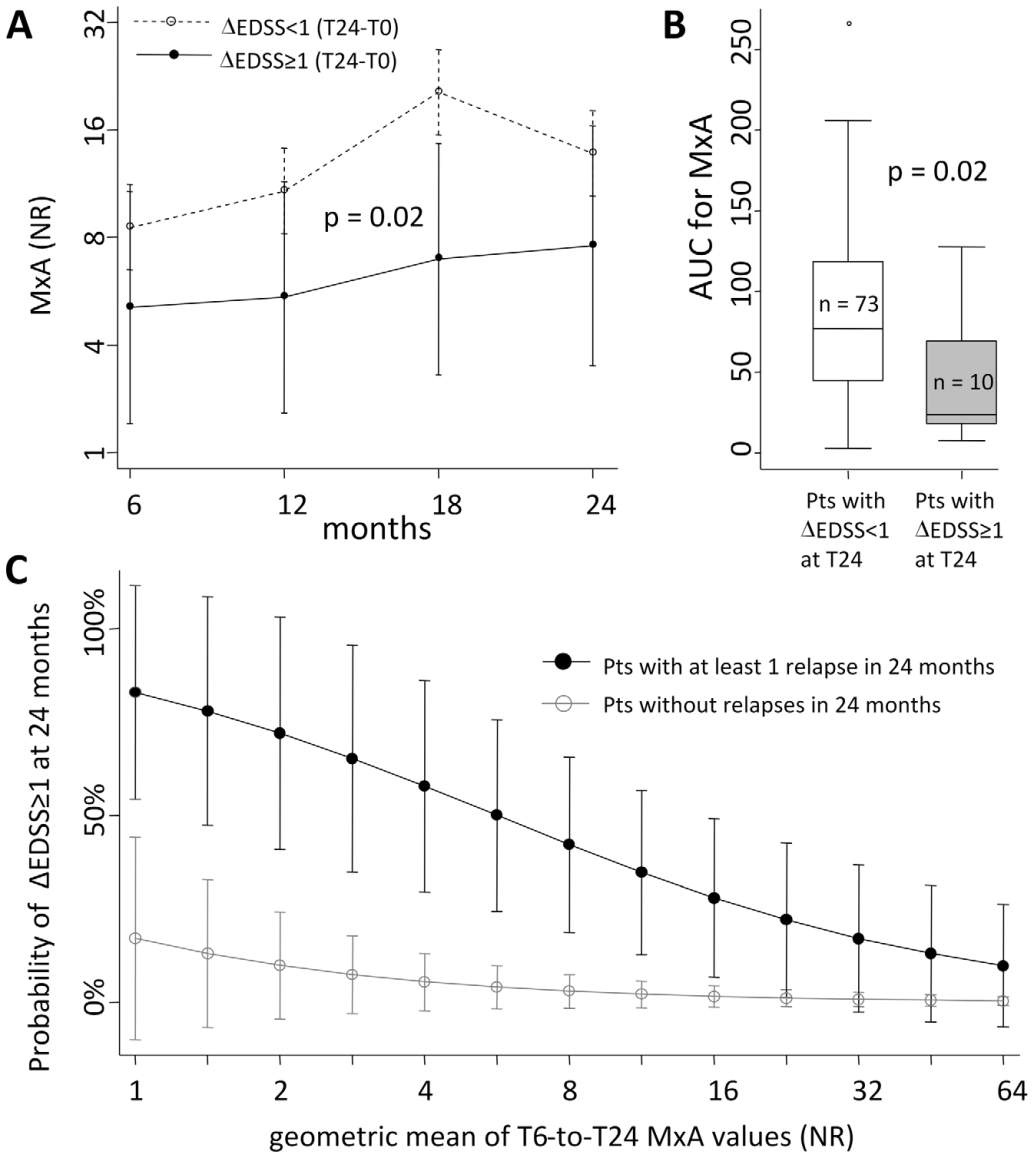
Average MxA as a potential marker of disability progression. Comparison of the log_2_MxA values (A) and of the area under the curve for non-transformed MxA mRNA levels calculated over the first two years of treatment (B) between patients with or without at least 1-point EDSS increase in the same period. Predicted probability of 1-point EDSS increase after 2 years of treatment in patients with at least one relapse (filled circles) vs. relapse-free patients (clear circles) in the same period (C). In (A) 95% confidence intervals are shown, while the shown p-value refers to the main effect of the ANOVA factor “1-point EDSS increase” (the interaction with the “time-point” factor was non-significant). In (B) the median, interquartile range, and range are shown as box-and-whisker plot. AUC: area under the curve; MxA: myxovirus-resistance protein A; NR: normalization ratio; EDSS: Expanded Disability Status Scale.

However, the analysis was done considering MxA as a binary, dichotomic variable (MxA^+^ vs. MxA^−^), determined only according to MxA mRNA level variation across a conventional threshold, while the amount of MxA mRNA varies in a continuous fashion [25], [26]. Thereafter, by repeated measure analysis, we compared the MxA values that were obtained across multiple observations in the patients with or without disability progression (10 [12%] vs. 73 [88%] patients), finding that patients showing a 1-point EDSS increase after 2 years of treatment had a significantly lower level of MxA induction (Figure 3A). More importantly, this difference was independent of the type of IFNβ treatment. A similar result was obtained by calculating the area under the curve for MxA expression from baseline to T24, which resulted lower in patients with a 1-point increased EDSS after 2 years (Figure 3B). These observations led to the hypothesis that a higher level of MxA induction could be associated to a better therapeutic response to IFNβ, ultimately affecting the likelihood of EDSS worsening. Therefore, in order to verify to what extent a higher average MxA level could determine a reduced risk of 1-point EDSS increase, we studied the relation between the two variables by multivariable logistic regression. The best fitting model showed that each 1-unit increase in the “average” log_2_MxA levels (or, in other words, each doubling in the geometric mean of non-transformed MxA) predicts a reduction of 47% in the risk of 1-point EDSS increase (OR: 0.53, p = 0.02; CI: 0.30–0.92), and that the presence of at least one relapse in the first 2 years is a very strong independent predictor of EDSS worsening risk (OR 22.40, p = 0.001, CI:3.63–138.11). No other covariates, including the type of IFNβ treatment, basal EDSS, bioactivity profile, and IFNAR expression levels, had any significant impact on this risk. In particular, with this model, it was possible to calculate that relapsed patients with a geometric mean of MxA ≥16 NR have a probability of EDSS worsening significantly lower than 50%, which is similar to that calculated for patients without relapses (Figure 3C). A potential limitation of this cross-sectional analysis is that it may suffer from selection bias due to attrition, i.e. the patients dropping-out before T24, and in particular due to the 12 subjects who left the study because of reported disease worsening (Figure 1). While in principle we cannot completely exclude it, this seems unlikely, because if we evaluate the few MxA determinations performed in these patients before they dropped-out, their average values were higher than the proposed cut-off in only 2 of them (not shown).

## Discussion

This 3-year prospective observational study was primarily aimed to study IFNβ bioactivity in a real-life setting. The primary tool to define each patient IFNβ bioactivity status was the quantification of MxA mRNA induction, the most established marker of IFNβ bioactivity [27]. It can be performed by a simple and reproducible real-time PCR assay [28], easily transferred to the routine clinical practice [18].

We found that, after therapy initiation, MxA mRNA was stably induced to a similar extent by IFNβ-1a i.m., IFNβ-1a 44 µg s.c., and IFNβ-1b preparations. The analysis of MxA-induction in the individual patients, i.e. when the MxA mRNA level was below a cut-off, allowed us to conclude that 90/100 patients had a conserved IFNβ bioactivity, while 10/100 of patients were biological non-responders. The pattern and kinetics of antibody production was rather heterogeneous: in 8 of these 10 non-responder patients the absence of MxA induction was always accompanied by the presence of BAbs, while NAbs were above their cut-off in the majority but not in all tested samples. This partial discrepancy can be due to the well-known technical issues regarding the detection and quantification of IFNβ-induced NAbs that may lead to NAb status misclassification in as much as 30% of samples, even in reference laboratories [11]. Therefore, although NAbs may sometimes be undetected, our result confirms that anti-IFNβ antibodies are linked to IFNβ bioactivity loss. In the remaining 2 biological (and clinical) non-responder patients repeated MxA non-inductions were observed in total absence of anti-IFNβ antibodies. Despite Gilli et al. [21] reported the IFNAR receptor system as a possible determinant of bioactivity loss in patients with similar features, we did not find any peculiar alterations of IFNAR subunit/isoform mRNA expression in these individuals. Overall, in our study population, IFNAR mRNA modulation was not correlated to IFNβ bioactivity loss, and in the debate about the agonistic or antagonistic implication of the soluble isoform [29], [30], our results are in support of an agonistic role, because higher levels of IFNAR2.3 mRNA were associated to higher MxA values. An alternative explanation for the repeated absence of MxA induction in total absence of anti-IFNβ antibodies could also be non-compliance. Even among the 90 patients that were classified as biological responders there were samples intermittently resulting MxA-non-induced within a background of repeated MxA induction and no antibodies, nor IFNAR alterations. Because all these patients were treated with IFNβ-1a i.m., which is administered once a week, the most likely interpretation could be patients not correctly assuming their drug or failure to report the correct timing of drug injection more than a true, sporadic loss of bioactivity. The absent MxA mRNA induction was not due to a concurrent treatment with statins that were shown to reduce the in vitro production of MxA [31], because only one patient assumed a drug of the statin class within a time frame concomitant with a sample resulting MxA^−^BAb^−^NAb^−^, and only at the last point of his follow up. Another possible explanation could be a genetic alteration in the IFNβ response genes, such as single nucleotide polymorphisms or mutations in the MxA gene promoter or in other regulatory regions involving the MxA response [32], [33], whereas by sequencing, we could exclude genetic alterations in the regions involved in the interaction with the real-time PCR primers and probe. Finally, we found that a late restoration of lost IFNβ bioactivity can occasionally occur.

When analyzed at the group level, the bioactivity profiles were for the most part unlinked from the clinical disease activity, thus not confirming previous reports indicating that MxA quantification can be associated to disease activity [34]. Besides differences in the study designs (prospective vs. retrospective), this discrepancy may arise because the classification of IFNβ bioactivity loss as an all-or-nothing phenomenon (MxA^+^ vs. MxA^−^) may not be sensitive enough to be correlated to individual changes in clinical disease activity in a short time interval. In this context, it had also been previously reported by Malucchi et al. [26] in another retrospective study that there are samples with intermediate levels of MxA defined as “grey-zone” that can have a reduced, yet not completely lost bioactivity. Indeed, we also found samples in which MxA was slightly above the cut-off in presence of low/intermediate titers of NAbs, indicating that bioactivity can sometimes fluctuate between being completely lost and being partially reduced, but not absent. These data prompted us to analyze the IFNβ bioactivity also on a continuous quantitative scale, which might be more appropriate to represent the biological therapy course. Accordingly, we found that in the first 2 years of study period the average amount of MxA mRNA induction, considered on its quantitative scale, was increased in patients with a lower risk of having a 1-point EDSS increase, which is the EDSS variation considered significant in describing a disability progression in our study [23], [24]. In particular, the proportion of the risk of 2-year progression that could be attributed to MxA was independent from the presence of clinically apparent relapses, which, by themselves, contributed as expected to this risk. As a result, according to our prospective study, the patients with “average” MxA values above 16 NR in the first 2 years of treatment, even in presence of clinically apparent relapses, had an estimated probability of disability progression lower than 50%, which was similar to the low probability observed in relapse-free patients. These data indicated, for the first time, that the levels of MxA, even if predictive of the relapse rate [25], [34], are linked to a clinical measure of disability accumulation, which, in turn, is known to be predictive of long-term disability [24], [35]. If similar results will be confirmed by larger studies, an evaluation of MxA on a quantitative scale may prove an efficient tool for identifying patients at high risk of progression, in addition to the commonly employed MRI lesion load and relapse rate.

A number of questions may arise from these findings. Because it is obvious that not only the number but also the localization and the severity of relapses are crucial determinants of disability accumulation, the most likely explanation of our results could be that a persistently bioactive treatment, even if unable to suppress their occurrence, can reduce relapse severity by an extent that appears correlated to increasing average MxA levels. The quantitative monitoring of MxA may also be a more sensitive marker of the magnitude of IFNβ-mediated effects against the basal “background” of inflammatory activity, where a continuous, and not all-or-nothing anti-inflammatory effect seems more biologically plausible, and should ultimately lead to less severe damages. Another improvement is that the MxA assay can help the clinicians to overcome the NAb dilemma: on one hand, in fact, it is now widely recognized that NAbs have an impact on therapy success at the population level, and, indeed, NAbs appeared as the cause of MxA non-induction in the majority of our patients, even if sometimes they were low titered and underdetected; on the other hand, the consensus reached between the leading North American and European neurological societies after a decade of debates concluded that, at the individual-patient level, the interpretation of NAb measurements can be ambiguous, and that in cases of intermediate or low NAb titers additional information is needed, which can be provided by MxA bioactivity measurements [9]. Finally, inconsistent results regarding the same patient's NAb status were obtained in four reference laboratories in as much as 30% of the samples [11]. On the contrary, the MxA mRNA assay appears as a more practical and convenient assay, easily standardized for the routine practice [18], [28], and our results provide a reference value to link bioactivity, clinical course, and therapy.

Therefore, in conclusion, we propose the average MxA mRNA quantification as a reliable marker of the IFNβ long-term response, which encompasses the severity of relapses and disability, and can potentially stand as the primary tool to routinely monitor IFNβ therapy.

## Supporting Information

Figure S1 **Patients' disposition and dropouts during the 3-year study period.** Reported as adverse events are the following (comprising both drug-related and drug-independent clinical events): injection site reaction (1), thyroiditis (1), liver enzyme elevation (4), leucopenia (1), kidney stones (1), migraine (1), pregnancy (1), breast nodule (1), and breast cancer (1).
(PDF)

Figure S2 **Comparison of BAb level between healthy donors (HD) and multiple sclerosis patients before therapy initiation.** BAb: binding antibodies.
(PDF)

Figure S3 **MxA and anti-IFNβ antibodies in all samples of all patients.** Red lines indicate the cut-offs. MxA: myxovirus-resistance protein A; NR: normalization ratio; BAbs: binding antibodies; NAbs: neutralizing antibodies.
(PDF)

Table S1 **Baseline characteristics of the 100 patients considered in the analysis.** EDSS: Expanded Disability Status Scale; IFNβ: interferon beta.
(PDF)
