# Supplementary figures and images for: MxA mRNA Quantification and Disability Progression in Interferon Beta-Treated Multiple Sclerosis Patients
Source: PLoS One. 2014 Apr 14;9(4):e94794. doi: 10.1371/journal.pone.0094794 (PMC3986392; doi:10.1371/journal.pone.0094794)

# Figure S1

## Patients' disposition

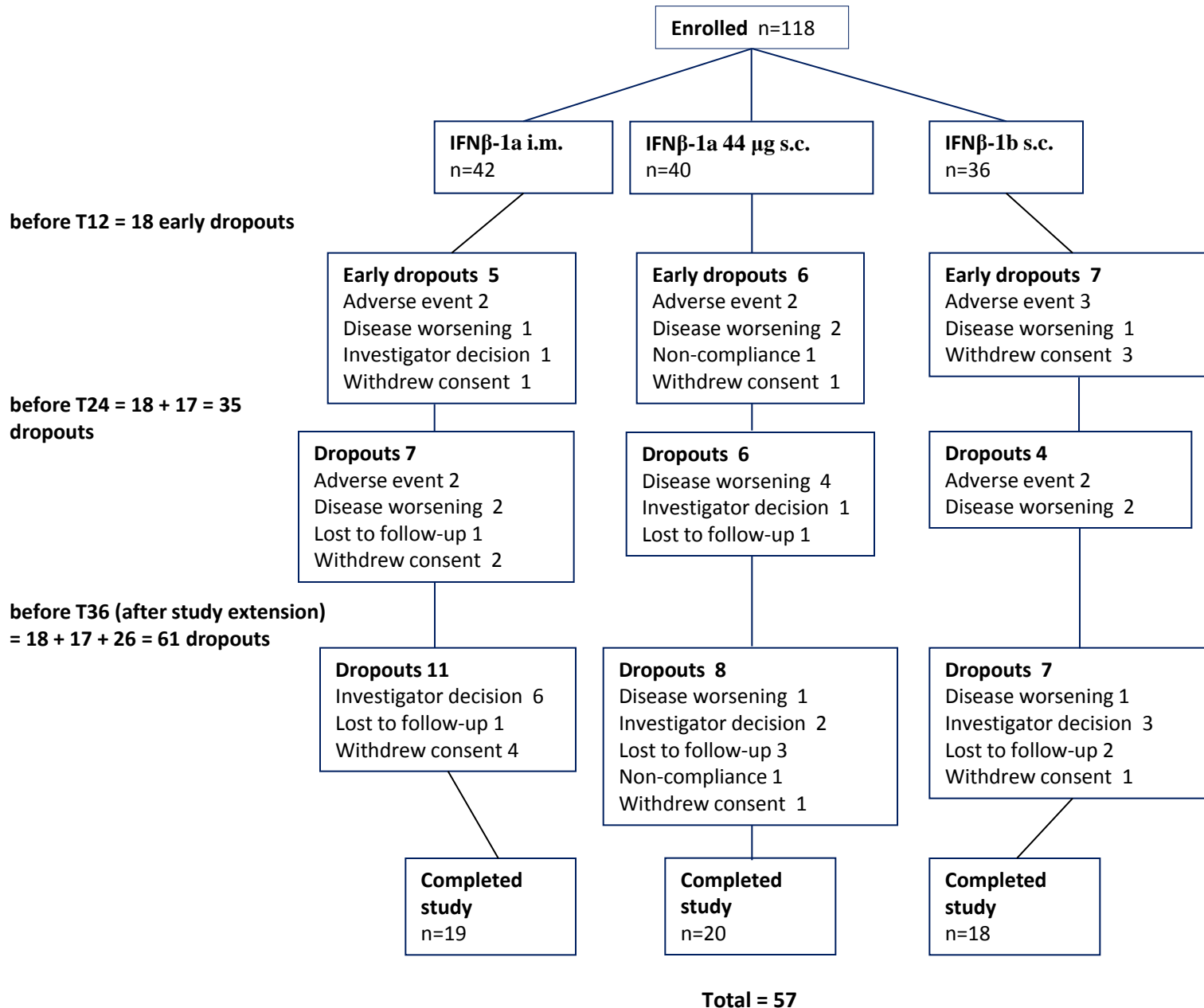

Supplement: Figure S1 — Patients' disposition and dropouts during the 3-year study period. Reported as adverse events are the following (comprising both drug-related and drug-independent clinical events): injection site reaction (1), thyroiditis (1), liver enzyme elevation (4), leucopenia (1), kidney stones (1), migraine (1), pregnancy (1), breast nodule (1), and breast cancer (1). (PDF) [file pone.0094794.s001.pdf]

**Figure S2**

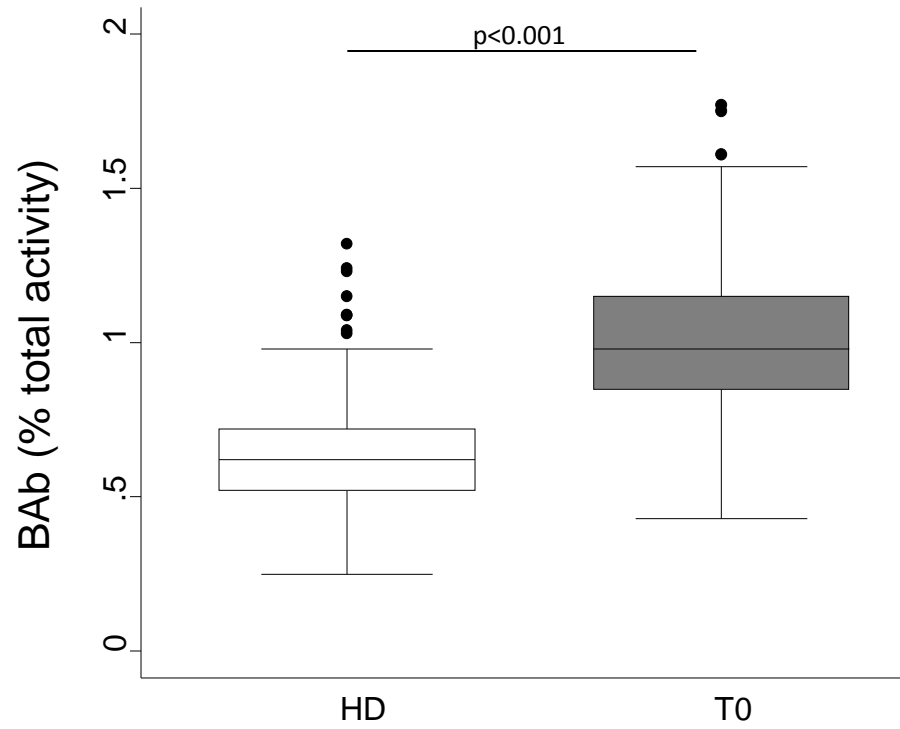

Supplement: Figure S2 — Comparison of BAb level between healthy donors (HD) and multiple sclerosis patients before therapy initiation. BAb: binding antibodies. (PDF) [file pone.0094794.s002.pdf]

**Figure S3**

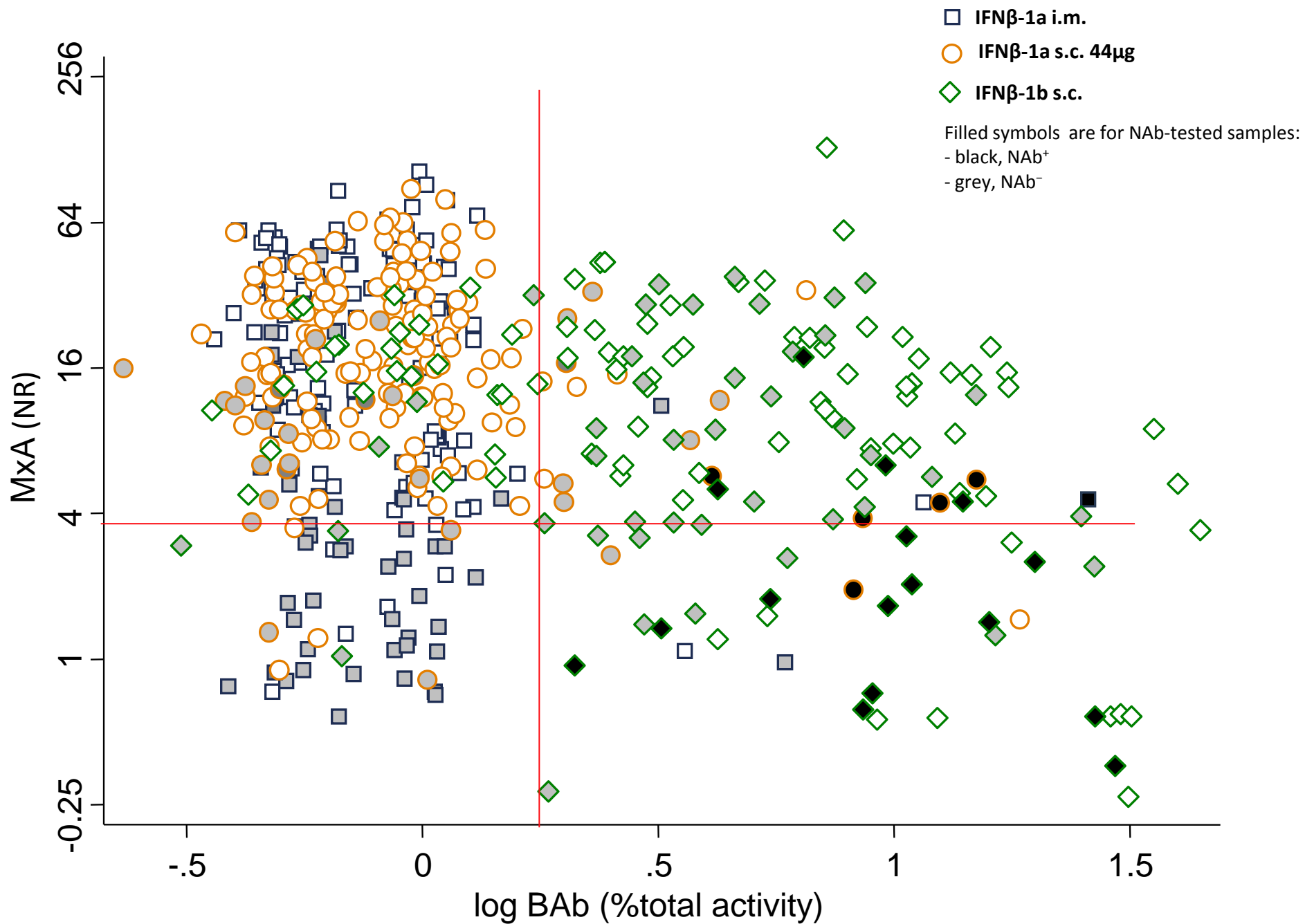

Supplement: Figure S3 — MxA and anti-IFNβ antibodies in all samples of all patients. Red lines indicate the cut-offs. MxA: myxovirus-resistance protein A; NR: normalization ratio; BAbs: binding antibodies; NAbs: neutralizing antibodies. (PDF) [file pone.0094794.s003.pdf]
