# Supplementary material for: MxA mRNA Quantification and Disability Progression in Interferon Beta-Treated Multiple Sclerosis Patients
Source: PLoS One. 2014 Apr 14;9(4):e94794. doi: 10.1371/journal.pone.0094794 (PMC3986392; doi:10.1371/journal.pone.0094794)
Supplement: Table S1 — Baseline characteristics of the 100 patients considered in the analysis. EDSS: Expanded Disability Status Scale; IFNβ: interferon beta. (PDF) [file pone.0094794.s004.pdf]

Table S1

Baseline characteristics of the 100 patients analyzed

	**Gender** (M:F)	**Age** median (range)*	**EDSS at T0** median (range)**
**IFNβ-1a i.m.**	10:27	38 (20-59)	1.5 (0-3.5)
**IFNβ-1a s.c.**	16:18	30 (18-58)	1.5 (0-3.5)
**IFNβ-1b s.c.**	6:23	45 (19-64)	2 (0-4.5)
**Total**	32:68	35 (18-64)	1.5 (0-4.5)

* p<0.01 at the Kruskall-Wallis test

**p<0.05 at the Kruskall-Wallis test
